# A privacy-preserving publicly verifiable quantum random number generator

**DOI:** 10.1038/s41598-024-61552-y

**Published:** 2024-05-17

**Authors:** Tanvirul Islam, Anindya Banerji, Chin Jia Boon, Wang Rui, Ayesha Reezwana, James A. Grieve, Rodrigo Piera, Alexander Ling

**Affiliations:** 1 Centre for Quantum Technologies, National University of Singapore, 3 Science Drive 2, Singapore, 117543 Singapore; 2 Quantum Research Centre, Technology Innovation Institute, Abu Dhabi, UAE; 3 Department of Physics, National University of Singapore, Blk S12, 2 Science Drive 3, Singapore, 117542 Singapore

**Keywords:** Quantum information, Quantum optics

## Abstract

Verifying the quality of a random number generator involves performing computationally intensive statistical tests on large data sets commonly in the range of gigabytes. Limitations on computing power can restrict an end-user’s ability to perform such verification. There are also random number-based applications where an honest user needs to publicly demonstrate that the random bits they are using pass the statistical tests without the bits being revealed. Here, we report the implementation of an entanglement-based protocol that allows a third party to publicly perform statistical tests without compromising the privacy of the random bits.

## Introduction

Generating random numbers that are private, secure, and have the statistical properties expected of a uniform randomness distribution is a crucial step for many computational tasks. For example, scientific simulations^1^, self-testing quantum systems^2^, randomized algorithms^3,4^, machine learning^5^, cryptography^6,7^, lottery, gambling, public tenders, computer games, utilize random numbers during initialization of the systems or during operation. Pseudo-random number generators (PRNG) based on algorithms can have good statistical properties resembling a uniform source, but strong long-range correlations exist in the output that may undermine the applications ^8^, or introduce security loopholes. This is because the seed to the PRNG is the only entropy in the system, and entropy cannot be increased by deterministic computation. Quantum random number generators (QRNG) ^9,10^ have been proposed as an alternative where entropy is extracted from a quantum mechanical process.

All random number generators, however, face two common problems. First, the user may lack sufficient computational capacity to perform the statistical tests^11–13^ needed to certify the quality of the randomness. Second, in public-facing applications, such as lottery or public tenders, the owner of the QRNG device may have to prove the statistical quality of the bits to public stakeholders before the bits are used. These problems require a solution which allows a user to publicly test their random bits without revealing them.

We propose that a publicly testable random number generator^14^ can be constructed if the device could generate correlated streams of random bits. A public tester performs arbitrary statistical tests on one of the bit streams to certify its randomness properties. By construction, this extends certification to the other output streams that are not shared with the verifier. Here, we only consider an honest user who wants to test the statistical quality of the generated random bits using external testing facilities. If the external testing facility acts as a certification authority then it allows the random bits from the user to be certified for public facing applications.

In this manuscript, we report the implementation of a QRNG using only a polarization-entangled photon pair source, and linear optics. This implementation satisfies the conditions of secrecy and public testability.

## Constructing a publicly verifiable QRNG

A publicly verifiable QRNG should have the following properties.

- **Property 1:** The source of the entropy is of quantum origin.
- **Property 2:** The quality of the QRNG output is publicly verifiable without compromising the secrecy of the final output bits.

In the following sections, we describe the steps for demonstrating a publicly verifiable QRNG.

### A protocol for publicly verifiable quantum random number generator

Property 1 is satisfied when an entanglement-based QRNG demonstrates that the source is producing a stream of entangled states and the random output is generated from the outcome of projective measurements on these entangled qubits. Here, the entanglement can be verified using Bell inequalities^15^. In our implementation below we use the CHSH inequality to ensure that Property 1 is satisfied.

A QRNG that produces a single stream of bits cannot be publicly verified without completely losing its secrecy. One needs a solution with at least two streams of bits, denoted $X_A$ and $X_B$, that are correlated in a way that publicly verifying the randomness of stream $X_A$ ensures the quality of the stream $X_B$. However, the protocol must ensure that their mutual information $I(X_A,X_B)=0$. Once achieved, the bit stream $X_B$ can serve as securely validated private randomness for public use. When this is achieved, the bit stream $X_B$ can be securely used as a publicly verified private randomness.

**Figure 1:**
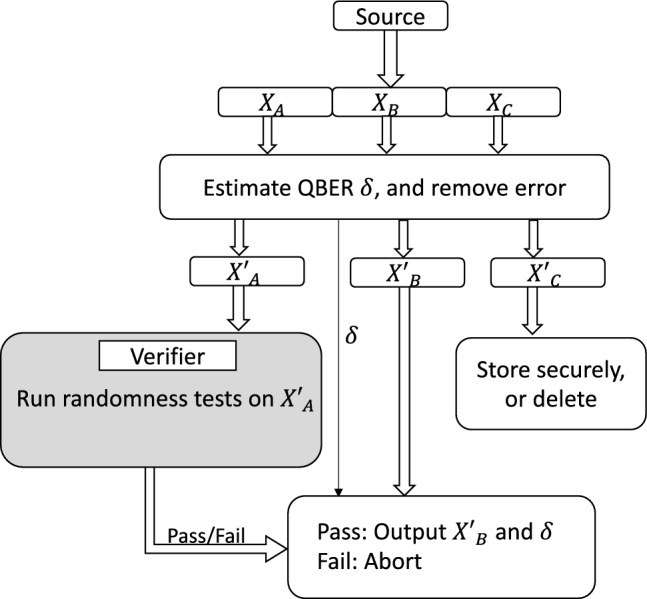
The QRNG outputs three correlated streams of random bits $X_A, X_B$ and $X_C$. Using them the quantum bit error rate (QBER), $\delta$ is estimated and the error triplet of bits are removed to generate $X'_A, X'_B$ and $X'_C$ . After this, $X'_A$ is sent to public verifier. $X'_C$ is stored securely or deleted. Verifier runs randomness tests on $X'_A$. If the test fails the protocol is aborted, else user outputs $X'_B$ and $\delta$. In this flowchart only the grey box is performed by the verifier, all other steps are performed by the user.

In our protocol, the QRNG produces three streams of random bits that are correlated. One of the bit streams is subjected to public randomness testing. As the streams are correlated this public randomness test verifies the quality of randomness in the other two unrevealed bit streams. This satisfies Property 2.

To achieve Property 1 and 2, we prepare a tripartite entangled state,

1 $$\begin{aligned} \vert \Phi _{ABC}\rangle =\frac{1}{2}(\vert 000\rangle - \vert 011\rangle +\vert 101\rangle - \vert 110\rangle ) \end{aligned}$$

This state exhibits the interesting property that performing a projective measurement in the computational basis on any one of the qubits projects the combined state of the other two qubits to either of two Bell states. As an example, if we measure qubit A in the computational basis the BC system is projected onto either Bell states, $\vert \Phi ^-_{BC}\rangle$ or $\vert \Psi ^-_{BC}\rangle$,

2 $$\begin{aligned} \vert \Phi _{ABC}\rangle =&\frac{1}{\sqrt{2}}\lbrace \vert 0\rangle \left( \frac{\vert 00\rangle - \vert 11\rangle }{\sqrt{2}}\right) +\vert 1\rangle \left( \frac{\vert 01\rangle - \vert 10\rangle }{\sqrt{2}}\right) \rbrace \end{aligned}$$

3 $$\begin{aligned} =&\frac{1}{\sqrt{2}}\lbrace \vert 0\rangle \vert \Phi ^-_{BC}\rangle +\vert 1\rangle \vert \Psi ^-_{BC}\rangle \rbrace \end{aligned}$$

Qubits prepared in a Bell state produce random outcomes when measured individually. The monogamy of entanglement^16^ ensures that this measurement outcome is not correlated to any outside system. Therefore, the outcome of the system *BC* cannot be predicted even if one has access to the outcome of *A*.

Consider a single copy of the state (1). We perform a projective measurement in the computational basis on the three subsystems of the state. Let $x_A, x_B$ and $x_C$ denote the outcomes of projective measurement of the three subsystems, *A*, *B* and *C* in the computational basis. They can be considered as bit valued random variables taking their values with probabilities from Table 1.

**Table 1:** Probability $p(x_A,x_B,x_C)$, of measurement outcomes $x_A$, $x_B$ and $x_C$ when each of the qubits A, B and C are subjected to projective measurement in the computational basis.

$p(x_A,x_B,x_C)$	$x_A$	$x_B$	$x_C$
1/4	0	0	0
1/4	0	1	1
1/4	1	0	1
1/4	1	1	0

If any one of the output columns is removed the remaining two columns show uniform distribution of two bits, indicating they are mutually independent. Outcomes that are not presented in the table have probability 0.

By construction of the state $\vert \Phi _{ABC}\rangle$ the outcomes always satisfy,

4 $$\begin{aligned} x_A \oplus x_B \oplus x_C = 0 \end{aligned}$$

where $\oplus$ is the addition modulo 2 operator.

Table 1 shows that the marginal probability distribution for $x_A$ is, $p(x_A=1)=p(x_A=0)=1/2$. Also, $x_B$ and $x_C$ have similar marginal distribution. Therefore, if we consider each of the three bits individually then they have maximal Shannon entropy,

5 $$\begin{aligned} H(x_A) = H(x_B) = H(x_C) = 1. \end{aligned}$$

From Table 1 we see that in the absence of knowledge of any one bit, the two other bits become completely uncorrelated with each other. That is, their marginal distribution can be factorized. Therefore, their mutual information is 0,

6 $$\begin{aligned} I(x_A,x_B) = I(x_B,x_C)= I(x_C,x_A) = 0. \end{aligned}$$

For random number generation, *n* copies of the state $\vert \Phi _{ABC}\rangle$ is prepared as in (1) and each of the three parts of the state is measured in the computational basis. The outcomes are recorded in bit strings $X_A, X_B$ and $X_C$ of lengths *n*. From our discussion so far, we see that each of the bit strings valued random variable $X_A, X_B$ and $X_C$ takes the value from strings in $\{0,1\}^n$ uniformly at random.

**Figure:**
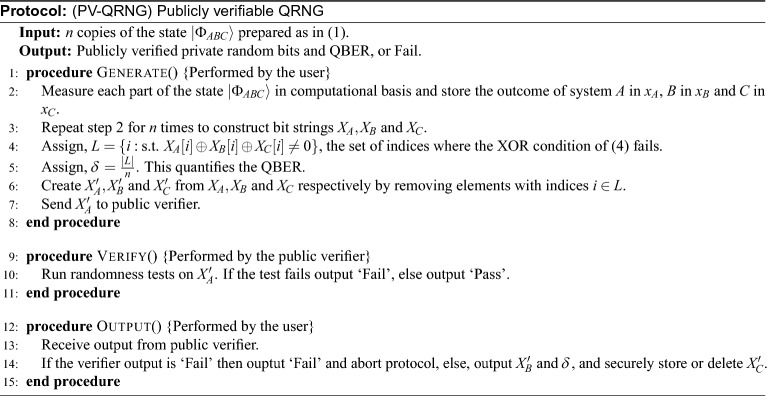
Protocol (PV-QRNG) Publicly verifiable QRNG

From the preparation, each copy of the state (1) is independent. Therefore, the condition (6) ensures that the random variables $X_A, X_B$ and $X_C$ are pairwise mutually independent. That is,

7 $$\begin{aligned} I(X_A,X_B) = I(X_B,X_C)= I(X_C,X_A) = 0. \end{aligned}$$

The string $X_A$ is provided to a public verifier that validates the string via statistical tests. If $X_A$ passes the randomness test, condition (5) ensures the quality of randomness of $X_B$ and $X_C$. As the verifier only has access to $X_A$, the condition (7) ensures that no information is leaked about $X_B$ or $X_C$. However, following (4), knowledge of any two bit strings would allow recovery of the third string. Therefore to satisfy Property 2, either $X_B$ or $X_C$ should remain inaccessible.

**Figure 2:**
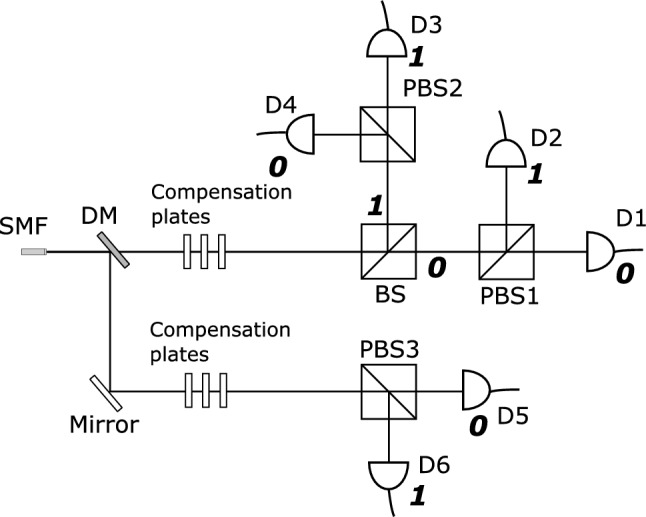
The detection setup. The boldfaced numbers represent the bit values encoded by the path of photons and define the bit streams $X_A$, $X_B$ and $X_C$. Entangled photons are launched from a single mode fiber (SMF) and separated according to wavelengths by dichroic mirror (DM). The polarization state of the photons in both paths are corrected by a stack of waveplates (Compensation plates). The output of the beam splitter (BS) generates $X_A$. Polarizing beam splitters PBS1 and PBS2 generate $X_B$. $X_C$ is generated by PBS3.

Imperfections in any practical implementation will lead to (4) not being always satisfied. Counting the number of events that do not meet the XOR condition (4) provides the quantum bit error rate (QBER). Removing the erroneous triplet of outcomes from $X_A, X_B$ and $X_C$ gives $X'_A, X'_B$ and $X'_C$ each of length *m* that satisfy,

8 $$\begin{aligned} X'_A \oplus X'_B \oplus X'_C = 0, \end{aligned}$$

where $\oplus$ denotes bit-wise addition modulo-2 operation.

At this point the user sends $X'_A$ to the public verifier for statistical randomness testing. If the verification fails then the user will discard the data and start over. If the verification succeeds then the user uses $X'_B$ as private randomness and securely stores or deletes $X'_C$. The presence of positive QBER (protocol output $\delta$) indicates information leakage to the environment. The user may use the QBER information to perform further randomness extraction to amplify the privacy (similar to privacy amplification^17^ in quantum key distribution).

The workflow of the protocol is depicted in Fig. 1 and the detailed steps are listed in Protocol PV-QRNG.

### The experimental setup

A source of non-degenerate entangled photon pairs, following the design demonstrated in [18], produces photon pairs in the Bell state $\vert \phi ^-\rangle = \frac{1}{\sqrt{2}} \left( \vert HH \rangle - \vert VV \rangle \right)$. Here $\vert H \rangle$ denotes horizontal polarization and $\vert V \rangle$ stands for vertical polarization. The photon pairs, coupled to a single mode fiber (SMF) are guided to the detection setup (see Fig. 2) where the signal photons ($\lambda = 780 nm$) are separated from the idler photons ($\lambda = 842 nm$) with the help of a dichroic mirror (DM). Stacks of quarter-half-quarter waveplates correct for the change in polarization state caused by birefringence in the SMF. The signal photons are directed to a polarizing beam splitter (PBS3) which performs a projection measurement in the *H*/*V* basis (horizontally polarized photons are transmitted, vertically polarized photons are reflected). The output ports of PBS3 define the bit $x_C$. If the photon is detected at D5, $x_C = 0$, and if it is detected at D6, $x_C = 1$. The idler photons encounter a non-polarizing beam splitter (BS). The photons are either transmitted or reflected at the BS with equal probability. This choice of path defines the bit $x_A$. Each output port of the BS consists of polarizing beam splitters (PBS1 and PBS2) and detectors. Acting similarly as PBS3, PBS1 and PBS2 are used to define the bit $x_B$. To illustrate, if the photon is transmitted at the BS and detected at D1, then $x_A = 0$ and $x_B = 0$. If it had been detected at D2, in that case, $x_A = 0$ and $x_B = 1$. However, if the photon was reflected at the BS and detected at D3, then $x_A = 1$ and $x_B = 1$. Similarly for a detection in D4, $x_A = 1$ and $x_B = 0$. Note here that the outcome labels for PBS2 have been flipped, which is akin to a local rotation of $\pi /2$ on the reflected path of the BS.

Due to polarization entanglement between the signal and idler photons, coincidence events are only expected to occur between the following detector pairs with equal probability: D1 and D5, D2 and D6, D3 and D5, D4 and D6. Together with $x_A$ determined from the choice of output port of BS, and flipping of the outcome labels of PBS2, the state in Eq. (1) can be realized. If the idler photons are transmitted at the BS, the photon pairs exist in state $\vert \phi _{BC}^-\rangle$, while if they are reflected at the BS, the local $\pi /2$ rotation implemented by flipping the outcome labels of PBS2, projects the photon pairs into state $\vert \psi _{BC}^-\rangle$.

#### Proof of entanglement

Generating a high fidelity Bell state is crucial to prepare the state (1) which preserves the secrecy of $X_B$ and $X_C$. Any QBER observed in the measurement outcome indicates the leakage of information to the environment and has to be taken care of in the privacy amplification step. (See, Section Privacy Analysis)

**Figure 3:**
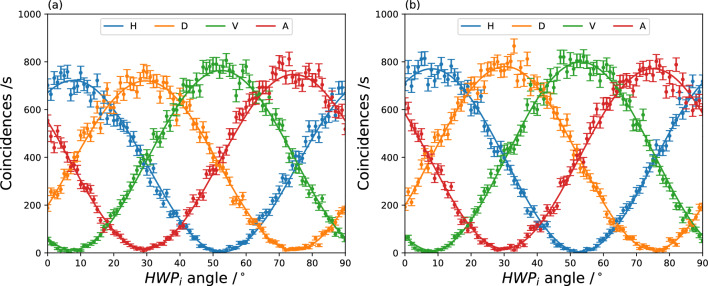
(**a**) Coincidences between (D1,D2) and D5, with visibilities of $0.988 \pm 0.006$, $0.971 \pm 0.009$, $0.967 \pm 0.009$, $0.96 \pm 0.01$ for the H, D, V and A bases respectively. (**b**) Coincidences between (D3,D4) and D5, with visibilities of $0.989 \pm 0.005$, $0.969 \pm 0.005$, $0.976 \pm 0.008$, $0.96 \pm 0.01$ for the H, D, V and A bases respectively. The visibilities for the coincidences between D1-4 and D6 (shown in supplementary material) are lower but are all above 0.93.

In the experimental setup (Fig. 2, halfwave plates were placed before BS and PBS3 to measure the visibility curves (Fig. 3) from which the CHSH^15^ values can be computed^18^. The detailed experimental setup for CHSH test and all the visibility curves are given in the supplementary information. The CHSH value for the state measured by systems (D1,D2) and (D5,D6) was $2.70 \pm 0.04$, while the value for the state measured by systems (D3,D4) and (D5,D6) was $2.72 \pm 0.04$.

#### Randomness testing results

**Figure 4:**
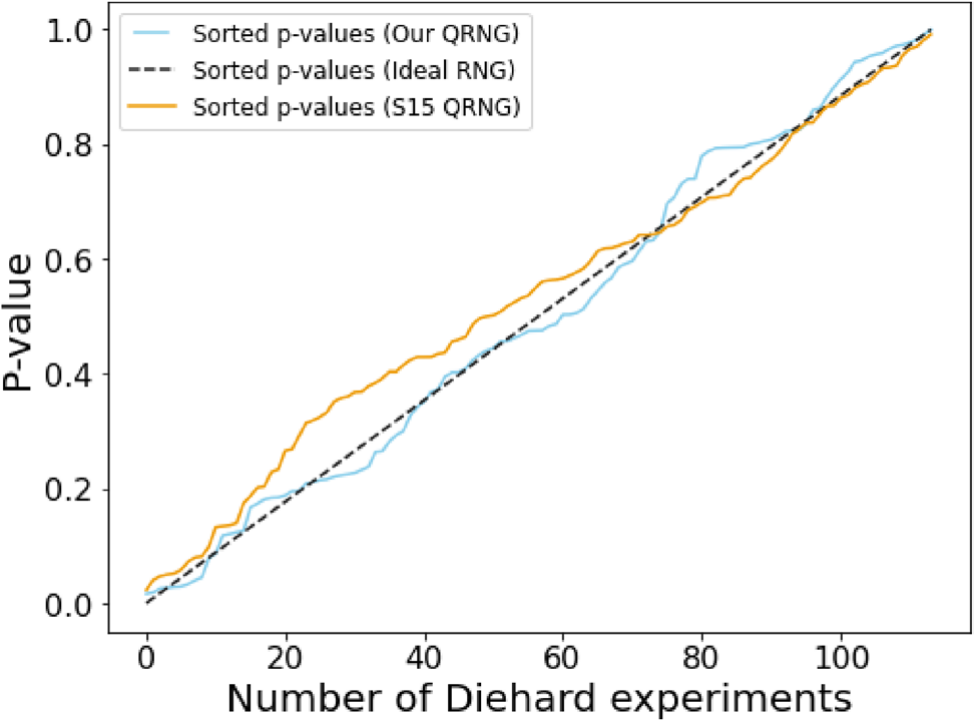
Result of the KS-test^20^. In this qualitative test *p* values obtained from the results of the dieharder test suite are sorted and plotted (blue line) against uniformly distributed values over the interval [0,1] (black dashed line). The orange line depicts the result from the tests run on an equal size of data obtained from QRNG1 ^21^ of S-Fifteen Instruments. The curves imply that our QRNG exhibits close to ideal expected performance.

We perform the statistical randomness test suite ‘dieharder’^19^ on random numbers generated using our implementation of Protocol PV-QRNG. This is to verify that the system is indeed generating good quality randomness. In dieharder, hundreds of hypothesis tests are performed on the input data set. If the input is random then the *p* values of these tests remain within the range [0.01, 0.99]. Although a thorough verification of randomness would require larger size of data and significantly more computational resource, our limited test shows that the data is very close to an ideal randomness source. The system is compatible for running extensive tests by any third party certification process. Using the computed *p* values Kolmogorov–Smirnov (KS) test^20^ is performed. Figure 4 shows a result for KS test that is performed on 1 MB of generated random bits. We run the same test on 1 MB of data from quantum random number generators by S-Fifteen Instruments^21^ and show it in the figure for comparison.

### Privacy analysis

In a practical setup, instead of getting the ideal state $\vert \Phi _{ABC}\rangle$ from (1), one might get a mixed state $\rho _{ABC}$ such that,

9 $$\begin{aligned} F(\Phi _{ABC},\rho _{ABC}) < 1-\varepsilon \end{aligned}$$

where, *F* is the fidelity, and $\varepsilon >0$.

This deviation from the ideal state will cause the the post measurement states to deviate from the ideal Bell states and show up as violation of the XOR condition (4). To be more precise, If we perform projective measurements on system *A* in the computational basis and keep the system *BC* untouched. The measurement operators can be defined as,

10 $$\begin{aligned} M^x_A = \vert x\rangle \langle x\vert \otimes I_{BC} \end{aligned}$$

for outcomes $x\in \{0,1\}$. And the post measurement states for system BC, would be

11 $$\begin{aligned} \rho ^x_{BC} = \frac{tr_A(M^x_A \rho _{ABC} M^{x\dagger }_A)}{tr(M^{x\dagger }_A M^x_A \rho _{ABC} )}. \end{aligned}$$

We analyze the privacy of the state $\rho ^0_{BC}$ and the argument for $\rho ^1_{BC}$ follows by symmetry.

The state $\rho ^0_{BC}$ can be written as,

12 $$\begin{aligned} \rho ^0_{BC} = (1-p)\vert \Phi ^-_{BC}\rangle \langle \Phi ^-_{BC}\vert + \frac{p}{4}I_{BC} \end{aligned}$$

where with probability *p* instead of getting the maximally entangled state $\vert \Phi ^-_{BC}\rangle$ we get a maximally mixed state.

Now, if we measure systems B and C of $\rho ^0_{BC}$ in computational basis then with probability *p*/2 the outcome would not match the expected outcome from $\vert \Phi ^-_{BC}\rangle$. Therefore, $\text {QBER}=p/2$. We can estimate *p* from the experimental measurements.

A non-zero QBER can be interpreted as information leakage out of the *BC* system. We can purify the state $\rho ^0_{BC}$ with environment *E* to get the purified state, $\vert \phi _{BCE}\rangle$, where

13 $$\begin{aligned} tr_E(\vert \phi ^0_{BCE}\rangle ) = \rho ^0_{BC}. \end{aligned}$$

From Protocol PV-QRNG we see that the output private bits are generated from system B. Thus, to estimate the number of private random bits that can be extracted from this system we can use the privacy analysis of an entanglement based quantum key distribution system between system B and C. With the exception, that the error correction step is perform by the user who has access to both B and C systems’ outcomes. Therefore, there is no leakage due to error correction. Moreover, the user can compute the population mean using the whole data set and does not have to reveal any public subset. All we need to estimate is the information leakage into the the environment E that reduces the privacy of the local outcomes of system B and C. Applying tight finite key analysis ^22^ in this scenario, we get that, from an output of length *n* of the Protocol PV-QRNG, at least $n(1-h(QBER))$ private random bits can be extracted, where *h* is the binary entropy function. This matches the asymptotic limit because for large *n* (for example, $n\approx 10^6$) the finite size effect is negligible.

## Discussion and future direction

We have presented a QRNG source where the source stream can be subjected to public statistical randomness testing without compromising the secrecy of the final output bits. Any change in detector efficiencies can be locally checked before sending out for public randomness testing. This allows the user to remove statistical bias in the bit strings to avoid information leakage. Along with robust miniaturized polarization entangled photon-pair sources, this setup can be built into a publicly verifiable QRNG source as a commercial off-the-shelf (COTS) product. Additionally, our entanglement based design can be extended to operate as a source device-independent^9^ publicly verifiable and auditable QRNG.

### Supplementary Information

Supplementary Figures.

## Data Availability

Data underlying the results presented in this paper are not publicly available at this time but may be obtained from the corresponding author upon reasonable request. The supplementary information file contains additional analysis of the data.
