# Supplementary material for: A privacy-preserving publicly verifiable quantum random number generator
Source: Sci Rep. 2024 May 17;14:11337. doi: 10.1038/s41598-024-61552-y (PMC11101663; doi:10.1038/s41598-024-61552-y)
Supplement: Supplementary file 1 — Supplementary Figures. [file 41598_2024_61552_MOESM1_ESM.pdf]

Supplementary Material: A privacy-preserving publicly verifiable quantum random number generator

Tanvirul Islam,[1] Anindya Banerji,[1] Chin Jia Boon,[1] Wang Rui,[1] Ayesha Reezwana,[1] James A. Grieve,[1,2] Rodrigo Piera,[2] and Alexander Ling[1,3]

[1] *Centre for Quantum Technologies, National University of Singapore, 3 Science Drive 2, 117543 Singapore*
[2] *Quantum Research Centre, Technology Innovation Institute, Abu Dhabi, United Arab Emirates*
[3] *Department of Physics, National University of Singapore, Blk S12, 2 Science Drive 3, 117542 Singapore*

I. VISIBILITY MEASUREMENT

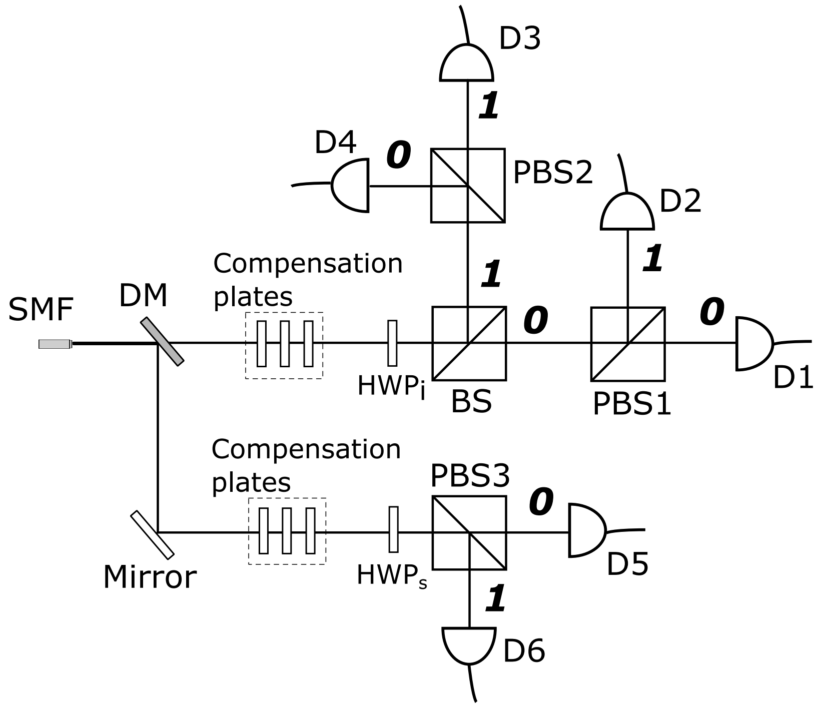

FIG. S1. The detection setup used to implement the QRNG protocol (reproduced from the main text) . The input to the SMF was a target state of $|\Phi^-\rangle = \frac{1}{\sqrt{2}}(|HH\rangle - |VV\rangle)$.

The visibility curves in Figure S2 were measured after placing halfwave plates, HWP_i and HWP_s, after the compensation plates. When the fast axis of HWP_s is set to be parallel to the optical table, detectors D1 and D3 measure the H basis while detectors D2 and D4 measure the V basis. When the fast axis of HWP_s is set to 22.5° from the plane parallel of the optical table, detectors D1 and D3 measure the D basis while detectors D2 and D4 measure the A basis. For each setting, HWP_i was rotated 90° with the coincidence counts between detectors measured every 1°.

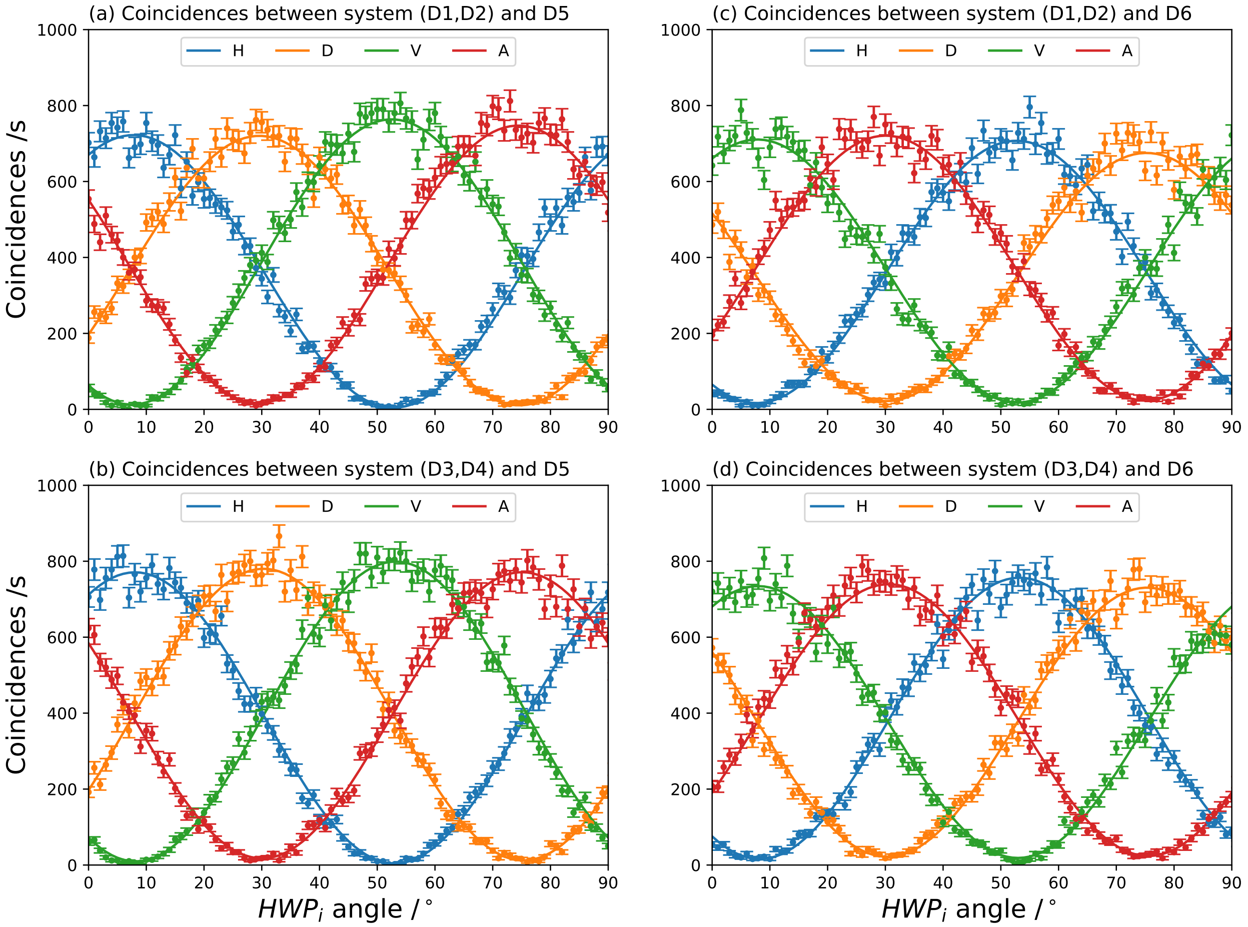

FIG. S2. Full results from the visibility measurement. The values reported below are the visibilities for the H, D, V and A bases for the respective detectors. (a) Coincidences between (D1,D2) and D5, with visibilities of $0.988 \pm 0.006, 0.971 \pm 0.009, 0.967 \pm 0.009, 0.96 \pm 0.01$; (b) Coincidences between (D3,D4) and D5, with visibilities of $0.989 \pm 0.005, 0.969 \pm 0.005, 0.976 \pm 0.008, 0.96 \pm 0.01$; (c) Coincidences between (D1,D2) and D6, with visibilities of $0.95 \pm 0.01, 0.94 \pm 0.01, 0.95 \pm 0.01, 0.94 \pm 0.01$; (d) Coincidences between (D3,D4) and D5, with visibilities of $0.96 \pm 0.01, 0.94 \pm 0.01, 0.95 \pm 0.01, 0.94 \pm 0.01$.
